# Assessing seasonality of travel distance to harm reduction service providers among persons who inject drugs

**DOI:** 10.1186/s12954-015-0081-y

**Published:** 2015-10-12

**Authors:** Sean T. Allen, Monica S. Ruiz, Amira Roess, Jeff Jones

**Affiliations:** Department of Prevention and Community Health, Milken Institute School of Public Health at The George Washington University, 950 New Hampshire Ave, Suite 300, Washington, DC 20052 USA; Department of Health Policy and Management, Jiann-Ping Hsu College of Public Health at Georgia Southern University, PO Box 8015, Statesboro, GA 30460 USA

## Abstract

**Background:**

Prior research has examined access to syringe exchange program (SEP) services among persons who inject drugs (PWID), but no research has been conducted to evaluate variations in SEP access based on season. This is an important gap in the literature given that seasonal weather patterns and inclement weather may affect SEP service utilization. The purpose of this research is to examine differences in access to SEPs by season among PWID in the District of Columbia (DC).

**Findings:**

A geometric point distance estimation technique was applied to records from a DC SEP that operated from 1996 to 2011. We calculated the walking distance (via sidewalks) from the centroid point of zip code of home residence to the exchange site where PWID presented for services. Analysis of variance (ANOVA) was used to examine differences in walking distance measures by season. Differences in mean walking distance measures were statistically significant between winter and spring with PWID traveling approximately 2.88 and 2.77 miles, respectively, to access the SEP during these seasons.

**Conclusions:**

The results of this study suggest that seasonal differences in SEP accessibility may exist between winter and spring. PWID may benefit from harm reduction providers adapting their SEP operations to provide a greater diversity of exchange locations during seasons in which inclement weather may negatively influence engagement with SEPs. Increasing the number of exchange locations based on season may help resolve unmet needs among injectors.

## Background

Syringe exchange programs (SEPs) are cost-effective, decrease the incidence of HIV among persons who inject drugs (PWID), and have not been shown to increase drug use, crime, or presence of discarded syringes in neighborhoods [1–5]. SEPs may also provide referrals to other services (e.g., housing, drug treatment, etc.) that may facilitate substance use cessation. The efficacy of SEPs is partially dependent on their accessibility. A study conducted in Baltimore, MD, found that injectors who resided in the same zip code as that of where they exchanged syringes were more likely to engage multiple times with the SEP than their counterparts who resided in a different zip code than where they accessed services [6]. Research has also shown that PWID who reside in close proximity to SEPs are more likely to access services consistently and are less likely to share injection equipment [7, 8].

Research conducted among the Philadelphia PWID population examined the spatial relationships between areas of relevance to injectors (e.g., where substances are purchased/used and home residence) [9]. This study found that the distance between injectors’ home residence and SEP exchange site was approximately 2.5 miles. Another study conducted among the District of Columbia (DC) PWID population found that injectors traveled approximately 2.75 miles to access services [10].

Notably, no study has examined if differences in SEP access exist among PWID populations based on seasonality. This is an important gap in the literature given that many PWID walk to engage with SEPs [10] and that they may be forced to resolve their need for SEP services in the contexts of extreme seasonal weather patterns that create transportation and SEP access barriers. The purpose of this research is to extend our knowledge of SEP access via two primary aims: (1) to quantify the approximate distance between home residence and SEP exchange site by season and (2) to examine seasonal differences in SEP access among DC injectors. We hypothesized that seasonal differences would exist with persons traveling shorter distances to access the SEP in winter.

## Findings

### Methods

Exchange records from a SEP that operated in DC from 1996 to 2011 were used to calculate the mean walking distance (via sidewalks) PWID traveled to access harm reduction services by season. At the time of registration, each client was asked a series of questions that ascertained their HIV status, zip code of home residence, injection practices, and other sociodemographic information. The dataset also included a record for each exchange event, including the location of where it took place. Because the purpose of this research was to understand SEP access among DC PWID, instances where a person reported a zip code of home residence outside of the District or at a post office box zip code (which may represent a location of convenience rather than a space near the participant’s home) were excluded. These exclusion criteria mirrored those used in a similar study of SEP accessibility [10].

Because home residence data were limited to zip code of home residence, a geometric point distance estimation technique was used to calculate the estimated walking distance between the zip code of home residence and exchange location. This technique has been used in other research that examined SEP access among injectors [10]. Briefly, distance measures are calculated using the geometric centroid (i.e., the geometric center) of a given unit of analysis (e.g., a zip code) with the assumption that all data pertaining to the unit of analysis have a common origin at the centroid point [11, 12].

All exchange locations were coded using Google Maps [13]. Map data of zip codes in the USA were downloaded from the United States Census Bureau [14] and imported to ArcMap v10.2.1. ArcMap was used to calculate the latitude and longitude coordinates of the centroid point of each zip code. These data points were then merged in Microsoft Excel. A SAS macro was used to quantify the walking distance between the centroid point of each zip code of home residence and exchange location.

Walking distance measures were analyzed by season; however, to achieve a more balanced study design, exchange data were only included in the analyses if there was a complete calendar year of exchanges available. This analytic decision was made because the SEP closed in February 2011 and few exchanges occurred during 2011. A variable was created denoting the approximate meteorological season of each exchange. Following the operationalizations created by the National Oceanic and Atmospheric Association (NOAA), seasons were defined as follows: March, April, and May were coded as spring; June, July, and August were coded as summer; September, October, and November were coded as fall; and December, January, and February were coded as winter [15]. Analysis of variance (ANOVA) was used to test for differences in walking distance measures by season. The George Washington University Institutional Review Board approved this study (IRB# 111421).

### Results

The dataset included records for 12,094 unique PWID who accessed SEP services from 1996 to 2011. Among these records, 54.3 % (*n* = 6571) had registration data that included zip code of home residence. In order to make the analyses more generalizable, some records were excluded due to small sample sizes and/or missing data; more specifically, persons who identified as transgender (*n* = 15), who had missing gender data (*n* = 104), who identified as a race/ethnicity other than African American/Black or Caucasian/White (*n* = 84), and who had missing race/ethnicity data (*n* = 30) were excluded. These exclusions resulted in a preliminary analytic sample consisting of exchange records from 6638 PWID. Among these persons, 87.8 % (*n* = 5593) reported a DC zip code of home residence; however, only 76.9 % of the records for DC PWID included exchange location data that was viable for distance estimation (i.e., the location(s) of the exchanges could be matched to a specific location). The registration data that were not viable for geocoding stemmed from limitations in how data were recorded at the SEP (i.e., locations that were coded as “Various Sites” or “Unidentified” were not able to be geocoded). Of the remaining 4300 PWID, 84.6 % (*n* = 3638) reported a zip code of home residence in DC that was a geographical zip code (i.e., not a post office box zip code). These 3638 persons and their collective 33,959 total exchange records formed the analytic sample for these analyses. Demographic and substance use profile data of the analytic sample are summarized in Tables 1 and 2.

**Table 1:** Descriptive statistics of the analytic sample (*n* = 3,638)

Variable		% of sample
Gender	Male	74.7
	Female	25.3
Race	African American/Black	96.6
	White	3.4
Housing status	Not marginally housed	27.4
	Marginally housed	62.4
	Missing housing data	10.2
Engagement in a drug treatment program	Never in a drug treatment program	49.8
	Previously in a drug treatment program	50.2
Employment status	Unemployed	75.2
	Employed part time	4.6
	Employed full time	9.6
	Missing employment data	10.5
Education level	Did not graduate high school	23.5
	Graduated high school (no college)	53.5
	Graduated high school (some college, no degree)	9.5
	Graduated from college	3.2
	Missing education data	10.3

**Table 2:** Analytic sample substance use measures (*n* = 3,638)

Substance	Reported use	% of sample
Heroin	Did not report use	12.4
	Reported use	87.6
Skin popping	Did not report use	57.5
	Reported use	42.5
Cocaine	Did not report use	66.1
	Reported use	34.0
Speedball	Did not report use	43.5
	Reported use	56.5

The walking distance measures remained relatively constant across all seasons. There were statistically significant differences (*p* < 0.05) in the walking distance measures between spring and winter (2.88 and 2.77 miles, respectively). No other statistically significant differences existed by season. These data are summarized in Tables 3 and 4.

**Table 3:** Mean walking distance by season

Season	Exchanges used in distance calculations	Mean walking distance (miles)	Standard deviation	Range
Spring	8,478	2.88	2.07	11.4
Summer	8,694	2.85	1.99	11.4
Fall	9,273	2.83	1.94	11.4
Winter	7,514	2.77	1.98	11.9

**Table 4:** ANOVA comparisons of mean walking distance measures by season

Season	Spring	Summer	Fall
Summer	0.6089		
Fall	0.2839	0.9508	
Winter	0.0027	0.0938	0.2549

## Discussion

The results of this research extend previous work [10] by providing evidence of seasonal differences in SEP accessibility among DC PWID. The fact that the walking distance measures were significantly different between spring and winter may be explained by seasonal variations in weather. PWID who reside in locations further from the SEP may be less likely to access services during periods of inclement weather. This may explain why the winter access measure was lower than spring (i.e., the winter measure reflected a greater proportion of exchange records from PWID who resided in close proximity to the SEP).

The results of this study fill a gap in the public health literature by documenting seasonal differences in one measure of SEP access among PWID. Although the magnitude of the difference in walking distance measures was small between winter and spring, it remains an important finding. These results may be used by SEP stakeholders during discussions pertaining to best practices for SEP service delivery strategies and, more specifically, how to serve the needs of PWID populations during periods of inclement weather (e.g., increasing the number of exchange locations, offering home delivery, etc.). These findings may also be used to guide discussions with policymakers about how the intersection of structural factors (e.g., buffer zone policies that restrict where SEPs may legally operate) that affect SEP access and seasonal impediments to SEP utilization may contribute to a risk environment that is not supportive of the public health of PWID populations.

As noted in previous research [10], one limitation of the geometric point distance estimation method is that it assumes all persons who report a given unit of analysis (e.g., a zip code) reside at a geometric centroid point. Calculations that are more accurate would have been possible with complete home addresses; however, these data were not available. Another limitation of this research is that it is plausible PWID may commute from locations other than their homes (e.g., shooting galleries, locations of drug purchase) to access SEP services and may do so via public or private transportation. These findings may not be reflective of PWID who travel from other locations to access the SEP and/or who do so via public or private transportation. An additional limitation of this research pertains to generalizability. These analyses are reflective of DC PWID and may not necessarily reflect seasonal patterns in SEP access among PWID populations in other locations. This is a notable limitation given that climatic variations based on location may impose differing SEP access impediments (e.g., hurricanes, extreme heat/cold, snowfall, tornadoes, etc.) in different seasons. Lastly, a large number of registrations were missing zip code of home residence data. This is likely due to the SEP’s focus on provision of sterile injection equipment rather than uniform and complete data collection.

## Conclusion

In conclusion, these results highlight seasonal differences in SEP accessibility. Inclement weather during winter months may serve as an impediment to service utilization. Harm reduction providers adapting their SEP operations such that services are delivered in more diverse locations during periods of inclement weather when persons may be less inclined to commute to exchange sites may help resolve unmet needs among injectors.

## Abbreviations

ANOVA: analysis of variance
DC: District of Columbia
PWID: persons who inject drugs
SEPs: syringe exchange programs
